# An Nd^3+^-Sensitized Upconversion Fluorescent Sensor for Epirubicin Detection

**DOI:** 10.3390/nano9121700

**Published:** 2019-11-28

**Authors:** Jingwen Mo, Long Shen, Qian Xu, Jiaying Zeng, Jingjie Sha, Tao Hu, Kedong Bi, Yunfei Chen

**Affiliations:** 1 Jiangsu Key Laboratory for Design & Manufacture of Micro/Nano Biomedical Instruments and School of Mechanical Engineering, Southeast University, Nanjing 210096, China; 220180361@seu.edu.cn (L.S.); major212@seu.edu.cn (J.S.); hutao@seu.edu.cn (T.H.); kedongbi@seu.edu.cn (K.B.); 2 School of Public Health, Southeast University, Nanjing 210009, China; 52tt1995@163.com

**Keywords:** upconversion, lanthanide doped nanoparticles, fluorescence, sensor, epirubicin

## Abstract

We describe here an Nd^3+^-sensitized upconversion fluorescent sensor for epirubicin (EPI) detection in aqueous solutions under 808 nm laser excitation. The upconversion fluorescence of nanoparticles is effectively quenched in the presence of EPI via a fluorescence resonance energy transfer mechanism. The dynamic quenching constant was 2.10 × 10^4^ M^−1^. Normalized fluorescence intensity increased linearly as the EPI concentration was raised from 0.09 μM to 189.66 μM and the fluorometric detection limit was 0.05 μM. The sensing method was simple, fast, and low-cost and was able to be applied to determine the levels of EPI in urine with spike recoveries from 97.5% to 102.6%. Another important feature of the proposed fluorescent sensor is that it holds a promising potential for in vivo imaging and detection due to its distinctive properties such as weak autofluorescence, low heating effect, and high light penetration depth.

## 1. Introduction

Epirubicin (EPI), an anthracycline antibiotic, is a highly effective antineoplastic agent, and is widely used for the treatment of various cancers including breast cancer, hepatocellular carcinoma, gastric cancer, lymphoma, and leukemia [1,2,3,4,5,6,7]. At an appropriate level, it can achieve the maximum therapeutic effect with minimal toxicity. However, the accumulation of EPI in the human body can be highly toxic and could have severe adverse effects, such as cardiotoxicity, myelosuppression, and hypoalbuminemia, potentially causing irreversible damage to organs [4,8,9,10]. To optimize cancer therapy, sensitive and selective detections of EPI are required to monitor the EPI concentration in biological samples.

Traditional techniques for the detection of EPI in biological fluids include liquid chromatography (LC), capillary electrophoresis (CE) and electrochemical methods [11]. Liquid chromatography generally requires pretreatment of biological samples using solid phase or liquid–liquid extractions and is coupled with additional detection approaches including ultraviolet (UV), fluorometry, or mass spectrometry (MS) [12,13,14,15,16,17,18,19,20,21]. LC-based methods, which benefit from an enriched target concentration after extractions, are very sensitive and accurate. Capillary electrophoresis is an alternative separation technique and is commonly coupled with laser-induced-fluorescence detection (LIF) [22,23,24]. However, these detection strategies are usually costly, time-consuming, or involve sophisticated instruments. Electrochemical sensors convert the interactions between the target analyte, EPI, and electrodes into an electrical signal and have fast response time and relatively high sensitivity [25,26,27,28,29]. Moreover, the sensitivity and selectivity can be further improved by modifying the electrode surface using carbon nanotubes, nanoparticles, and DNA [30,31,32,33,34]. Nonetheless, issues such as non-specific binding, the reproducibility of measurements, long term stability of sensors, and complex processes required to modify the electrode still need to be addressed [35].

In recent years, fluorescent sensors have attracted a great deal of attention because they are relatively simple, fast, low cost, and sensitive and have been widely used in various biological applications [36,37,38,39,40]. However, as far as we know, no fluorescent sensors have been reported for EPI detection. Available fluorescence probes, including quantum dots and organic dyes have been developed for detecting other anthracycline antibiotics including doxorubicin, daunorubicin, idarubicin, and mitoxantrone [41,42,43,44,45,46,47]. These down-conversion fluorescence probes generally require the use of ultraviolet or visible light as the excitation source, which causes many problems, such as a low signal to noise ratio, possible damage to cells and organs, and a low light penetration depth. In contrast, lanthanide doped upconversion nanoparticles (UCNPs), which transform the near infrared irradiation to a shorter wavelength fluorescence (e.g., visible light), circumvent these aforementioned problems and have inherently distinctive advantages, including weak autofluorescence, good biocompatibility, a narrow emission peak, and high photochemical stability [48,49,50,51]. To advance the in vivo applications of UCNPs, the excitation wavelength has been altered from 980 nm to ~800 nm by doping Nd^3+^ ions, which minimize the laser-induced heating effect due to significant reductions in the absorption coefficient of water [52,53,54,55].

**Figure 1:**
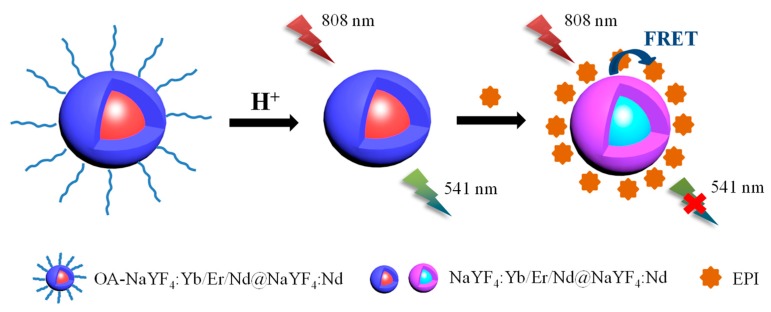
Schematic representation of the design principle of the Nd^3+^-upconversion nanoparticles (UCNPs) for epirubicin (EPI) detection. Water-dispersible UCNPs are obtained by removing hydrophobic oleate ligands from the surface of oleic acid coated UCNPs through acid treatment. When EPI is introduced, the UCNPs will attract EPI by electrostatic interactions. The green upconversion emission of the UCNPs is quenched due to the fluorescence resonance energy transfer (FRET) process between EPI and the UCNPS.

In this work, we have designed and synthesized a ligand-free Nd^3+^-sensitized upconversion fluorescence sensor (NaYF_4_:Yb/Er/Nd@NaYF_4_:Nd) for measurements of EPI under 808 nm laser excitation. UCNPs acts as an energy donor while EPI works as an energy acceptor. Owing to the overlap between the emission band of UCNPs and the absorption band of EPI, the fluorescence intensity of UCNPs can be effectively quenched in the presence of EPI through a fluorescence resonance energy transfer (FRET) process (Figure 1). The proposed sensing method is operationally convenient, fast, and cost effective and its overall performance is comparable to conventional methods employing sophisticated equipment or requiring tedious sensor preparations. The application of the sensing system was demonstrated by detecting the EPI levels in urine.

## 2. Materials and Methods 

### 2.1. Chemicals and Reagents

Yttrium(III) acetate hydrate (99.9%), ytterbium(III) acetate hydrate (99.9%), erbium(III) acetate hydrate (99.9%), neodymium(III) acetate hydrate (99.9%), ammonium fluoride (NH_4_F, 98+%), 1-octadecene (ODE, 90%), oleic acid (OA, 90%), epirubicin hydrochloride (EPI, 90+%), and L-cysteine (L-Cys, 97%) were purchased from Sigma-Aldrich. Histidine (His, 99%) and glucose (Glu, 99%) were bought from Macklin. Phosphate buffered saline (PBS) was obtained from HyClone. Hydrogen chloride (HCl), sodium hydroxide (NaOH), potassium chloride (KCl), sodium chlorate (NaCl), calcium chloride (CaCl_2_,) magnesium chloride (MgCl_2_), aluminum chloride (AlCl_3_), and L-glycine (L-Gly) were supplied from the China National Pharmaceutical Group Corporation. Cyanidin 3-glucoside, doxorubicin, and daunorubicin were purchased from Shanghai yuanye Bio-Technology company. All chemicals and reagents were analytically pure and used without any further purification.

### 2.2. Characterization

Upconversion fluorescence spectra were recorded on an Edinburgh F96S luminescence spectrometer (Shanghai Lengguang Technology Co., China) at room temperature with the excitation of an external 0–2 W adjustable continuous wave semiconductor laser at 808 nm (Changchun Laser Optoelectronics Technology Co., China). The morphology and size of the UCNPs were determined by transmission electron microscopy (TEM, Tecnai G2, FEI Company, Hillsboro OR, USA). The crystal structure and the phase purity of the UCNPs were obtained using an X-ray diffractometer (XRD, Bruker D8-Discover, Karlsruhe, Germany). Ultraviolet-visible (UV-Vis) absorption spectra were recorded on a UV-Vis spectrophotometer (Shimadzu UV-1780, Kyoto, Japan). The Fourier transform infrared (FTIR) spectra were performed using a Nicole iS10 FTIR spectrometer (Thermo Fisher Scientific, Waltham, USA) from samples in KBr pellets. 

### 2.3. Synthesis of Oleic Acid (OA)-Coated NaYF_4_:Yb/Er/Nd

NaYF_4_:Yb/Er/Nd nanoparticles were synthesized using the co-precipitation method [56]. Following the typical procedure, 1 mmol rare-earth acetates (Y/Yb/Er/Nd = 77:20:2:1) were mixed with oleic acid (10 mL) and 1-octadecene (15 mL) in a 100 mL flask under vigorous stirring. The solution was heated to 156 °C under argon protection and maintained for 1 h to form the lanthanide oleate complexes and remove the residual water and oxygen. Then, the solution was cooled down to 50 °C. A methanol solution (20 mL) containing NH_4_F (4 mmol) and NaOH (2.5 mmol) was added into the flask and incubated for 30 min. The temperature was increased to 70 °C to remove the methanol and then heated to 305 °C under an argon atmosphere for 1.5 h, before being cooled down to room temperature. The resulting nanoparticles were precipitated from the solution by the addition of ethanol and collected via centrifugation at 6000 rpm. The precipitated nanoparticles were repeatedly washed with ethanol and cyclohexane and finally redispersed in cyclohexane.

### 2.4. Synthesis of OA-Coated Core@shell NaYF_4_:Yb/Er/Nd@NaYF_4_:Nd

By using the synthesized NaYF_4_:Yb/Er/Nd core nanoparticles as seeds, an NaYF_4_:Nd layer was grown through the epitaxial growth method [56]. A total of 0.5 mmol of rare-earth acetates (Y/Nd = 70:30) were mixed with oleic acid (10 mL) and 1-octadecene (15 mL) in a flask. The solution was heated to 156 °C under argon flow for 1 h with magnetic stirring and then cooled to 50 °C. Core nanoparticles (1 mmol) in cyclohexane (10 mL) were added along with a methanol solution (20 mL) containing NH_4_F (4 mmol) and NaOH (2.5 mmol) into the flask and stirred at 50 °C for 30 min. The temperature was increased to 70 °C to remove the methanol and then heated to 305 °C under an argon atmosphere for 1.5 h, before being cooled down to room temperature. The resulting nanoparticles were precipitated out by the addition of ethanol, collected by centrifugation, washed with ethanol and cyclohexane, and finally redispersed in cyclohexane by ultrasonication.

### 2.5. Synthesis of Ligand-Free NaYF_4_:Yb/Er/Nd@NaYF_4_:Nd

Ligand-free core@shell UNCPs were prepared using acid treatment. Oleic acid (OA)-coated core@shell nanoparticles (0.2 mmol) in cyclohexane (1 mL) were added with ethanol. The mixture was centrifuged at 6000 rpm to precipitate the nanoparticles. The collected nanoparticles were added with 0.1 M HCl solution (1 mL). The reaction was performed with ultrasonication for 1 h at 45 °C. During the reaction, the carboxylate groups of the OA ligand were protonated to form OA. Next, the solution was mixed with 0.1 mL cyclohexane to remove OA by extraction with cyclohexane. Subsequently, the ligand-free UCNPs were collected by centrifugation at 14,000 rpm for 0.5 h. The product was washed repeatedly with deionized water and redispersed in deionized water for further experiments.

### 2.6. Optimization of Experimental Conditions

The effect of pH on the fluorescence intensity with and without EPI (26.73 μM) was investigated using solutions containing ligand-free core@shell UCNPs (0.4 M) with pH values adjusted from 4 to 8 by adding HCl or NaOH. Under an optimized pH, the influence of the ligand-free core@shell UCNP concentration on the quenching efficiency was explored with the addition of EPI (26.73 μM). The optimal reaction time was determined under the optimized ligand-free core@shell UCNP concentration in PBS.

### 2.7. Assay Conditions for the detection of EPI

A stock solution of 137.94 mM EPI was prepared in deionized water and stored at 4 °C. Different concentrations of EPI were obtained by diluting the stock solution. The concentration of ligand-free core@shell UCNPs was adjusted to 0.6 M by adding PBS. For the fluorometric detection, various concentrations of EPI solutions were added into 3 mL of ligand-free core@shell UCNP (0.6 M) solution. To avoid a change in volume, each addition was 1.5 μL. The final concentration of EPI was varied from 0.09 μM to 258.63 μM. Reactions were performed for 10 min at room temperature before fluorescence spectrum measurements. 

### 2.8. Real Sample Preparation

The urine sample was collected from a healthy volunteer. The sample was diluted 10 fold before analysis and no other pretreatments were used.

## 3. Results and Discussion

### 3.1. Characterization of UCNPs

**Figure 2:**
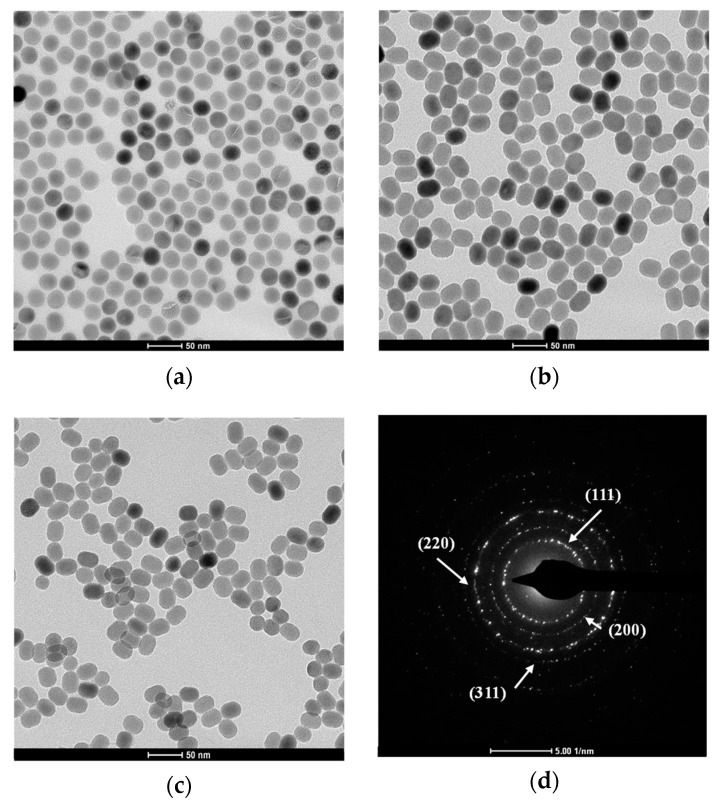
TEM images of (**a**) oleic acid (OA)-coated core UCNPs; (**b**) OA-coated core@shell UCNPs; (**c**) ligand-free core@shell UCNPs; (**d**) selected area electron diffraction (SAED) pattern of ligand-free core@shell UCNPs.

Prepared core (NaYF_4_:Yb/Er/Nd), core@shell (NaYF_4_:Yb/Er/Nd@NaYF_4_:Nd), and ligand-free core@shell (NaYF_4_:Yb/Er/Nd@NaYF_4_:Nd) UCNPs were analyzed by TEM. Figure 2a demonstrates that the synthesized core UCNPs are well-dispersed spherical particles and uniform in size with an average diameter of ~24.7 ± 1.7 nm (mean ± standard deviation). To enhance the upconversion fluorescence intensity, an external shell doped with Nd^3+^ was grown on the surface of the core UCNPs via the epitaxial growth method. Owing to an overall increase in the doping concentration of Nd^3+^, this active shell layer can enhance the harvesting of excitation energy. Additionally, the core-shell structure can minimize the surface quenching effect of the core and reduce cross relaxation between lanthanide ions [52]. As illustrated in Figure 2b, the resulting core@shell UCNPs were highly monodispersed elliptic particles with a length of 32.2 ± 1.4 nm and width of 25.1 ± 1.7 nm. The physical shape and size of these core@shell UCNPs was consistent with those of reported core@shell UCNPs synthesized using the same method [56]. To make core@shell UCNPs water-dispersible, the surface of the UCNPs was modified by removing oleate ligands. Figure 2c shows that the ligand-free core@shell UCNPs disperse well in water and retain the same narrow size distribution as the core@shell UCNPs. The successful removal of oleate ligands after acid treatment was also confirmed by Fourier-transform infrared spectroscopy. As shown in Figure S1, for the spectrum of core@shell UCNPs, peaks at 2927 and 2853 cm^−1^ are attributed to symmetric and asymmetric stretching vibrations of C-H, and peaks at 1556 and 1464 cm^−1^ are caused by symmetric and asymmetric stretching of COO-respectively, which are all associated with oleate ligands [57]. After surface modifications, these characteristic bands almost all vanished.

**Figure 3:**
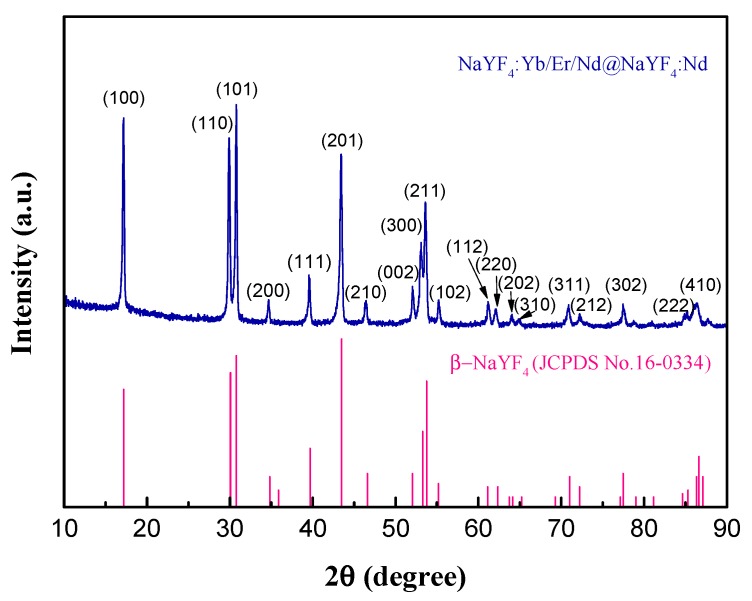
X-ray diffraction pattern of ligand-free core@shell UCNPs. The standard pattern of β-NaYF_4_ (JCPDS no. 16-0334) is also shown.

The crystal structure of the ligand-free core@shell UCNPs (denoted by Nd^3+^-UCNPs) was examined by X-ray diffraction (XRD), as shown in Figure 3. It was found that the peak positions and intensities were in good agreement with the calculated values of the pure hexagonal-phase structure β-NaYF_4_ nanocrystals (JCPDS no. 16-0334). In addition, the selected area electron diffraction pattern (SAED) of the Nd^3+^-UCNPs (Figure 2d) displays spotty polycrystalline diffraction rings, which correspond to the (111), (200), (220), and (311) planes of β-NaYF_4_ lattice. It is noted that β-phase NaYF_4_ is preferred over α-NaYF_4_, since it has a much stronger fluorescence intensity and better fluorescent thermal stability [58].

### 3.2. Principle of Detection

**Figure 4:**
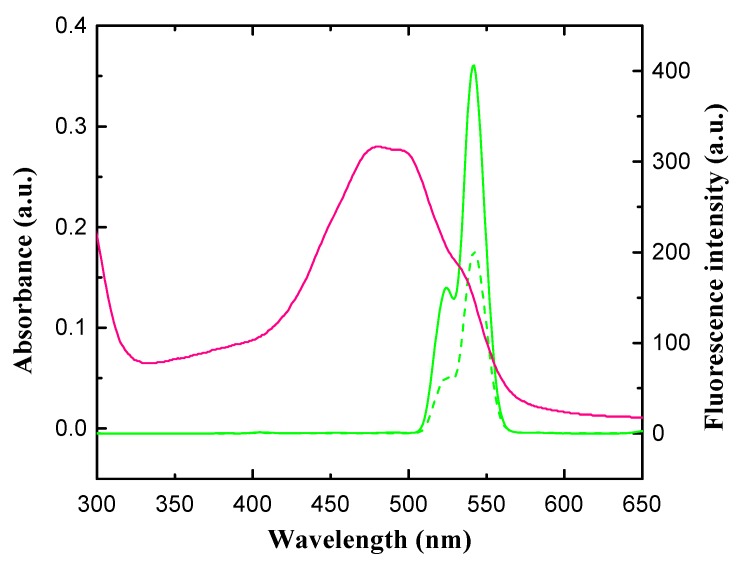
The emission spectrum of Nd^3+^-UCNPs without (green line) and with EPI (green dashed line), and the UV absorption of EPI at pH = 7.4 (red line).

The principle of the fluorometric detection of EPI using Nd^3+^-UCNPs is illustrated in Figure 1. The detection is based on the fluorescence resonance energy transfer process between Nd^3+^-UCNPs and EPI, where Nd^3+^-UCNPs works as an energy donor and EPI acts as an energy acceptor. Upon 808 nm excitation, the Nd^3+^ ions doped in the shell layer absorb and transfer the excitation energy to nearby Yb^3+^ ions in the nucleus and subsequently to the luminescent Er^3+^ center, which yields the upconversion emission. As shown in Figure 4, the Nd^3+^-UCNPs exhibit two emission bands in the green parts of the visible region. The emission bands at around 523 nm and 541 nm are due to ^2^H_11/2_-^4^I_15/2_, ^4^S_3/2_-^4^I_15/2_ transitions of Er^3+^, respectively [59]. The absorption spectrum of EPI (26.73 μM) in neutral pH conditions (pH = 7) was measured using a UV-Vis spectrophotometer. Figure 4 demonstrates that EPI has absorption peaks at 480 nm and 497 nm, which are close to the emission peaks of Nd^3+^-UCNPs. The absorption band of EPI (~400 nm–~600 nm) matched well with the emission band of Nd^3+^-UCNPs; therefore, they work as a donor and acceptor pair. Furthermore, the distance between the donor and the acceptor plays a critical role in affecting the rate of energy transfer [54]. It has been reported that the surface of Nd^3+^-UCNPs is negatively charged in neutral and alkaline solutions, while EPI is positively charged because of protonated amino nitrogen [60,61]. Therefore, EPI and Nd^3+^-UCNPs would interact via electrostatic attractions to form a new complex and quench the fluorescence intensity, as displayed in Figure 4. Notably, the maximum emission of Nd^3+^-UCNPs is located at ~541 nm. Therefore, the emission at 541 nm was used as the quantitative signal in the further experiments. 

### 3.3. Optimization of Experimental Conditions

To achieve high sensitivity and precision, several experimental conditions were optimized including the pH of the system, concentration of Nd^3+^-UCNPs, and reaction time. The effect of pH on the detection system was investigated with 0.4 M EPI (Figure S2). Considering that EPI may undergo hydrolysis in alkaline solutions (pH > 7.4), PBS (pH = 7.4) for the detection system was selected for further experiments to attain a low detection limit (Figure S2) [62].

**Figure 5:**
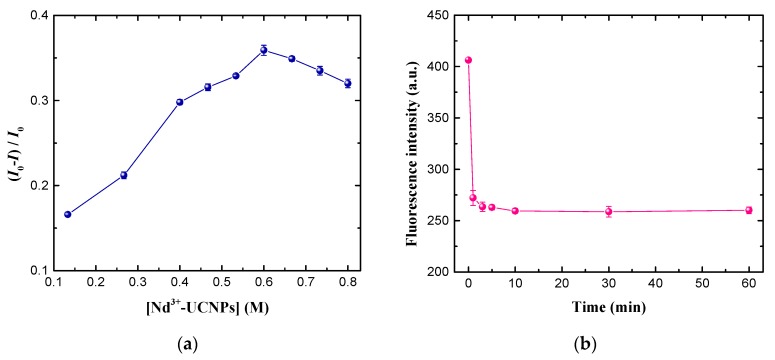
(**a**) Quenching efficiency, $(I_{0}-I)/I_{0}$, versus the concentration of Nd^3+^-UCNPs in phosphate buffered saline (PBS) ($I_{0}$ and I are the upconversion fluorescence intensity of Nd^3+^-UCNPs monitored at 541 nm in the absence and presence of 26.73 μM EPI, respectively); (**b**) Time-dependent upconversion fluorescence response of Nd^3+^-UCNPs in PBS with the addition of 26.73 μM EPI.

The effect of the concentration of Nd^3+^-UCNPs on the quenching efficiency was explored. The fluorescence intensity of Nd^3+^-UCNPs with and without the addition of EPI were denoted by I and $I_{0}$, respectively. Figure 5a demonstrates the quenching efficiency, computed by $(I_{0}-I)/I_{0}$, with the concentration of Nd^3+^-UCNPs varying from 0.13 to 0.8 M. It is seen that the quenching efficiency reaches the maximum when the concentration of Nd^3+^-UCNPs reaches ~0.6 M. Hence, the concentration of Nd^3+^-UCNPs was fixed at 0.6 M for the following experiments.

The reaction time also plays an important role in the detection. Figure 5b shows the temporal evolution of the quenched fluorescence intensity of Nd^3+^-UCNPs at 541 nm upon the addition of EPI (26.73 μM). The fluorescence intensity decreases sharply in the first several minutes, and reaches a steady state after a reaction time of 10 min. Furthermore, the fluorescence intensity remains stable for about 1 h, which demonstrates good stability of the detection system. Therefore, the reaction time was set to 10 min in all experiments.

### 3.4. Fluorometric Detection of EPI

**Figure 6:**
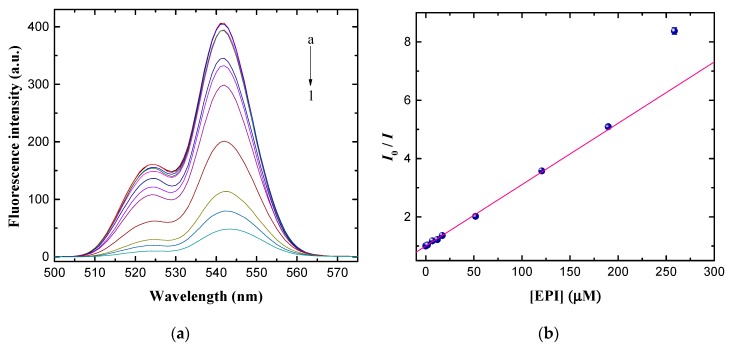
(**a**) The emission spectra of Nd^3+^-UCNPs after the addition of various concentrations of EPI, a–l: 0, 0.09, 0.17, 0.86, 1.72, 6.90, 12.07, 17.24, 51.73, 120.69, 189.66, and 258.63 μM in PBS; (**b**) Normalized fluorescence intensity, *I*_0_/*I*, plotted against EPI concentration (0.09–258.63 μM).

A series of fluorescence spectra of Nd^3+^-UCNPs in the presence of various concentrations of EPI were recorded under the optimized experimental conditions, as illustrated in Figure 6a. The fluorescence intensity decreases monotonously with the increase in EPI concentrations. Figure 6b plots the fluorescence intensity ratio, $I_{0}/I$, (monitored at 541 nm) against the concentration of EPI (0–258.63 μM). A good linear relationship occurs (R^2^ = 0.994) when the concentration of EPI is in the range of 0.09 μM to 189.66 μM. Quantified analysis can be performed using the Stern-Volmer equation, which is given by,

(1) $$I_{0}/I=1+K_{sv}\times [Q]$$

where $K_{sv}$ is the Stern-Volmer quenching constant and [Q] is the concentration of the quencher, EPI. $K_{sv}$ was computed to be 2.10 × 10^4^ M^−1^ using a linear least square fit. The $K_{sv}$ of the order 10^4^ demonstrates that EPI is an efficient quencher. The detection limit was determined to be 0.05 μM, which was calculated by 3σ/K, where σ represents the standard deviation of the blank signal and K is the slope of the linear curve. To compare with other methods, Table 1 summarizes the detection limit and the linear range of other techniques for EPI detection. The detection limit of this method is comparable to that of electrochemical methods [28,29,30,31], which is sufficiently sensitive for practical applications. Furthermore, this detection system has a wider linear range than that of other methods [13,16,20,22,28,29,30].

### 3.5. Selectivity for the Detection of EPI

**Figure 7:**
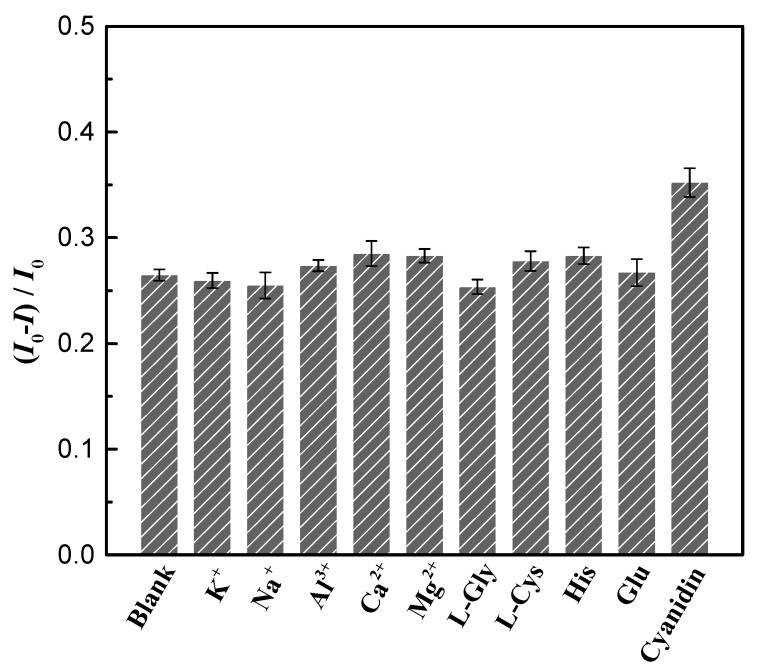
The relative fluorescence quenching efficiency of the detection system in the presence of other interfering molecules and ions in coexistence with EPI. $I_{0}$ represents the fluorescence intensity of Nd^3+^-UCNPs while I denotes the fluorescence intensity of Nd^3+^-UCNPs with the addition of EPI and other interfering species.

The interference of some metal ions and biomolecules that are normally present in biological fluids on the detection of EPI by the Nd^3+^-UCNPs probe was examined. As shown in Figure 7, except for 3 fold cyanidin 3-glucoside, the quenching efficiency when 17.24 μM EPI was added was not affected by a coexisting substance including 500 fold K^+^, 500 fold Na^+^, 500 fold Al^3+^, 300 fold Ca^2+^, 200 fold Mg^2+^, 1000 fold L-glycine, 500 fold L-cysteine, 500 fold histidine, or 300 fold glucose. The addition of cyanidin 3-glucoside would attenuate the upconversion fluorescence intensity, because cyanidin 3-glucoside has similar absorption spectra to that of EPI [63]. To gain a clear picture of the interference effect of cyanidin 3-glucoside, the quenching efficiency was measured with the concentration of cyanidin 3-glucoside varied from 0.1724 μM to 34.48 μM, as demonstrated in Figure S3a. It was found that the quenching efficiency was insensitive to variations in the concentration of cyanidin 3-glucoside when its concentration was low (less than 8.62 μM). Since normally the concentration of anthocyanin metabolites in urine is very low, their interference effects on the determination are minimal [63]. Even if a large anthocyanin dose is consumed, concentrations of anthocyanin metabolites in urine at 24 h are reduced to levels of ~0.01 μM [63]. In this case, their interference effects could be effectively eliminated by waiting sufficiently long time before the investigation. Furthermore, most potential interferences can be largely eliminated by simple dilution. Additionally, we performed experiments to investigate the interference effect in the presence of other anthracycline antibiotics including doxorubicin and daunorubicin. As shown in Figure S3b, the quenching efficiency was affected by the presence of doxorubicin or daunorubicin, especially when their concentrations were large. The current nanoprobe is limited to detecting EPI without the presence of some anthracycline antibiotics, whose molecular structure is similar to that of EPI. However, generally, EPI is not combined with other anthracycline antibiotics in actual treatment. To further improve the specificity, the UCNP surfaces would need to be modified with aptamer or molecularly printed polymers, which can interact specifically with EPI [30,64]. This will be investigated in future work.

### 3.6. Sample Analysis

For practical applications, the proposed Nd^3+^-UCNPs sensor was employed to detect EPI in human urine. The detection of EPI was performed by spiking a specified concentration of EPI in 3 mL urine sample containing Nd^3+^-UCNPs (0.6 M) using the standard addition method. As shown in Table 2, the recovery for the sample was in the range of 97.5–102.6%, with a relative standard deviation (RSD) from 4.7% to 6.1%. This result confirms the feasibility of using Nd^3+^-UCNPs for EPI detection in human urine samples. 

## 4. Conclusions

In summary, a simple and selective Nd^3+^-UCNPs fluorescent sensor for rapid and reliable detection of EPI in aqueous solutions has been proposed. Water-dispersible Nd^3+^-UCNPs are prepared by simply removing the oleic ligands from the core@shell UCNPs through acid treatment. The absorption of EPI onto the Nd^3+^-UCNP surface effectively quenches the upconversion fluorescence intensity via the FRET process. This sensing method is fast and sensitive and has a wide linear range. The interference from common ions and biomolecules is minimal. Urine sample testing demonstrates that the detection strategy is applicable for real sample analysis. Finally, we would like to point out that the Nd^3+^-UCNPs sensor is an excellent emitter with low autofluorescence and a high penetration depth to biological samples, which holds great potential for applications in biological and analytical fields such as in vivo imaging, sensing, and therapy.

## Figures and Tables

**Table 1:** Comparison of previous methods for EPI analysis. LOD, limit of detection; HPLC/UV, high performance liquid chromatography coupled with ultraviolet; HPLC/MS/MS, high performance liquid chromatography coupled with mass spectrometry; CE/LIF, capillary electrophoresis coupled with laser induced fluorescence; UCNP, upconversion nanoparticle.

Methods	LOD (μM)	Linear Range (μM)	Refs.
HPLC/Fluorometry	0.00129	0.0043–4.311	[13]
HPLC/UV	0.05	0.172–86.2	[16]
HPLC/MS/MS	0.00017	0.00034–0.0069	[20]
CE/LIF	0.017	0.086–0.862	[22]
Electrochemical/bare electrode	0.126	0.86–70.0	[29]
Electrochemical/nanoparticles	0.01	0.04–450	[31]
Electrochemical/carbon nanotube	0.02	0.05–10	[28]
Electrochemical/DNA	0.04	0.07–21	[30]
Fluorescence/Nd^3+^-UCNPs	0.05	0.09–189.66	This work

**Table 2:** Recovery experiments of EPI in urine samples.

Sample	Added (μM)	Found (μM)	Recovery (%, *n* = 5)	RSD (%, *n* = 5)
Urine 1	5	4.9	98.2	4.7
Urine 2	10	10.3	102.5	6.1
Urine 3	20	23.2	97.5	5.8

## Supplementary Materials

The following are available online at https://www.mdpi.com/2079-4991/9/12/1700/s1, Figure S1: FTIR spectra of (**a**) OA-coated core@shell UCNPs, and (**b**) ligand-free core@shell UCNPs, Figure S2: (**a**) Upconversion fluorescence responses of Nd^3+^-UCNPs in the absence and presence of 26.73 μM EPI at different pH values, (**b**) UV absorption spectra of EPI for a pH from 4 to 8, Figure S3: The fluorescence quenching efficiency of the detection system in the presence of (**a**) cyanidin 3-glucoside, (**b**) doxorubicin or daunorubicin in coexistence with EPI. *I*_0_ represents the fluorescence intensity of Nd^3+^-UCNPs while *I* denotes the fluorescence intensity of Nd^3+^-UCNPs with the addition of EPI (17.24 μM) and other interfering species.
