# Supplementary material for: An Nd3+-Sensitized Upconversion Fluorescent Sensor for Epirubicin Detection
Source: Nanomaterials (Basel). 2019 Nov 28;9(12):1700. doi: 10.3390/nano9121700 (PMC6955805; doi:10.3390/nano9121700)
Supplement: Supplementary file 1 [file nanomaterials-09-01700-s001.pdf]

nanomaterials

Supplementary Material

Article

An Nd^{3+}-Sensitized Upconversion Fluorescent Sensor for Epirubicin Detection

Jingwen Mo [1,*], Long Shen [1], Qian Xu [2], Jiaying Zeng [2,*], Jingjie Sha [1], Tao Hu [1], Kedong Bi [1] and Yunfei Chen [1,*]

[1] Jiangsu Key Laboratory for Design & Manufacture of Micro/Nano Biomedical Instruments and School of Mechanical Engineering, Southeast University, Nanjing 210096, P.R. China; 220180361@seu.edu.cn (L.S.); major212@seu.edu.cn (J.S.); hutao@seu.edu.cn (T.H.); kedongbi@seu.edu.cn (K.B.)

[2] School of Public Health, Southeast University, Nanjing 210009, P.R. China; 52tt1995@163.com (Q.X.)

* Correspondence: yunfeichen@seu.edu.cn (Y.C.); jwmo@seu.edu.cn (J.M.); jyzeng@seu.edu.cn (J.Z.)

The upconversion fluorescence intensities of Nd^{3+}-UCNPs in the absence and presence of EPI versus pH are illustrated in Figure S2 (a). It is seen that the fluorescence intensity of Nd^{3+}-UCNPs remains stable in pH 4–8. After adding EPI, the quenched fluorescence intensity remains almost unchanged when pH ≤ 6 and decreases as pH is further increased. The decrease is likely caused by the enhanced attraction between Nd^{3+}-UCNPs and EPI. EPI has an amino sugar moiety, which is positively charged because of protonated amino nitrogen [1]. The surface of Nd^{3+}-UCNPs is turned to be negatively charged when pH is higher than ~6 [2]. Due to strong electrostatic attractions, the distance between Nd^{3+}-UCNPs and EPI would be closer, which increases the energy transfer rate and facilitates the quenching [3]. Nonetheless, EPI is highly unstable in alkaline solutions and would undergo hydrolysis even at room temperature [4,5]. It is possible that the degradation of EPI may occur at pH = 8. We then investigate the absorption spectrum of EPI (26.73 μM) at different pHs. As shown in Figure S2 (b), the absorption spectrum of EPI is stable and has two peaks at 478 nm and 496 nm for pH between 4 and 7.4. However, the maximum absorbance peak is shifted to 508 nm at pH = 8, which is highly likely caused by the hydrolysis of EPI. To avoid this, PBS (pH = 7.4) of the detection system was selected for the following experiments.

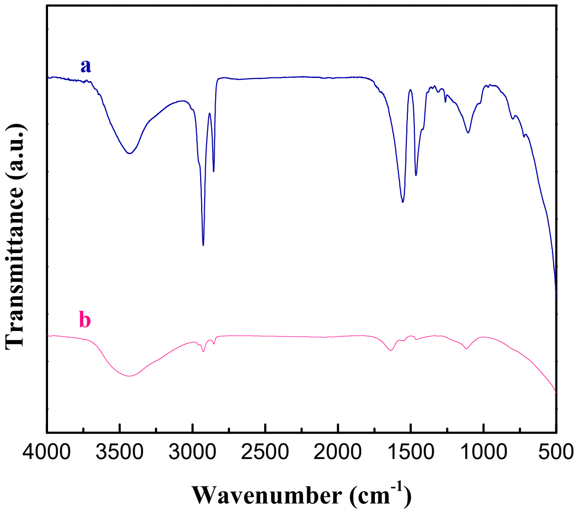

Figure S1. FTIR spectra of (**a**) OA-coated core@shell UCNPs, and (**b**) ligand-free core@shell UCNPs.

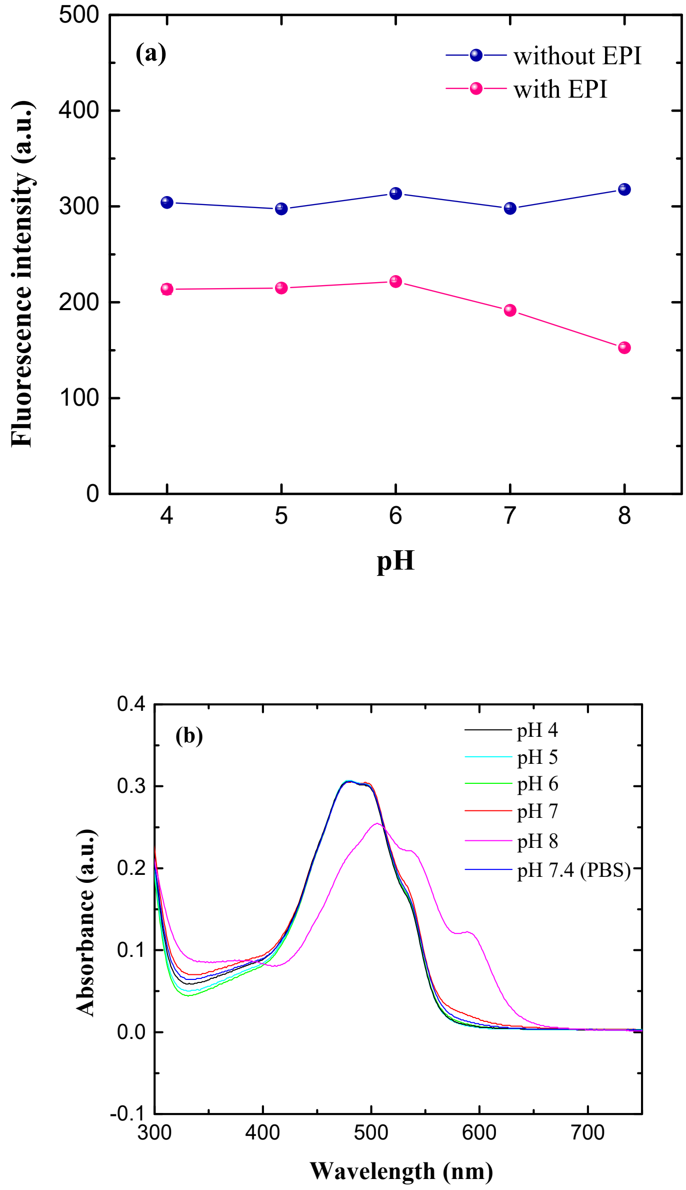

Figure S2. (**a**) Upconversion fluorescence responses of Nd^{3+}-UCNPs in the absence and presence of 26.73 μM EPI at different pH values, (**b**) UV absorption spectra of EPI for pH from 4 to 8.

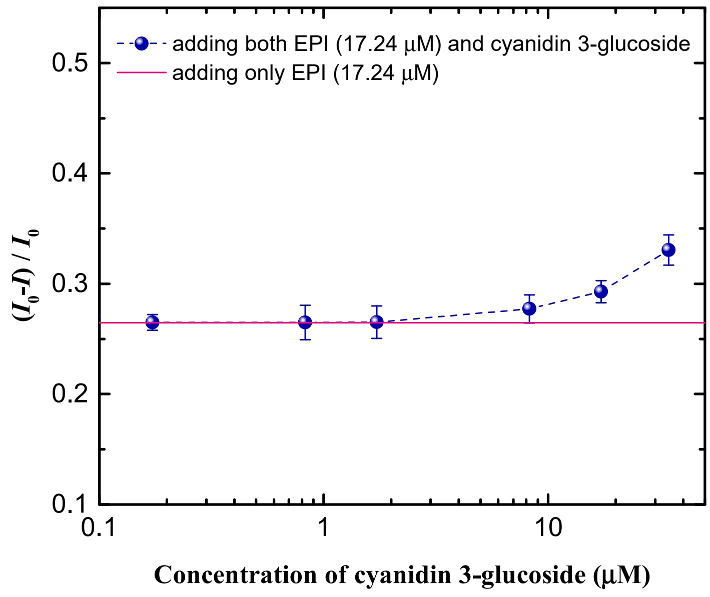

(**a**)

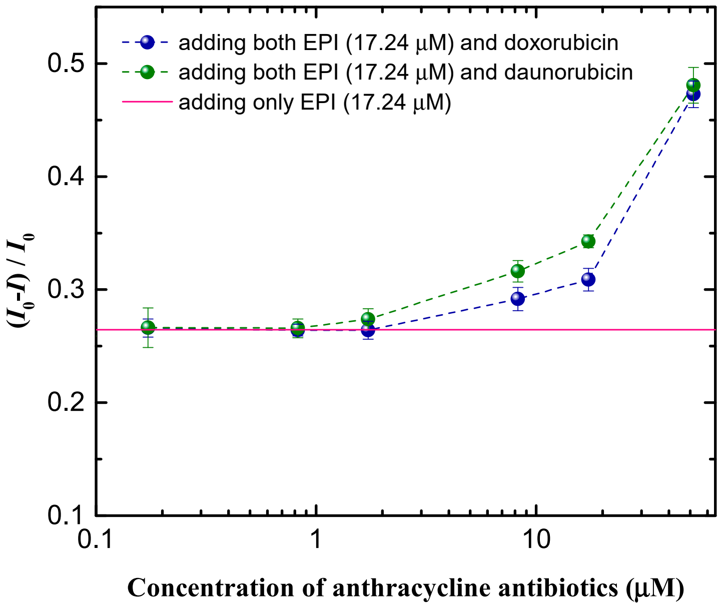

(**b**)

Figure S3. The fluorescence quenching efficiency of the detection system in the presence of (**a**) cyanidin 3-glucoside, (**b**) doxorubicin or daunorubicin in coexistence with EPI. I_0 represents the fluorescence intensity of Nd^{3+}-UCNPs while I denotes the fluorescence intensity of Nd^{3+}-UCNPs with the addition of EPI (17.24 μM) and other interfering species.

References

1. Ocak, I.; Kara, H.E.S. Phosphorescent detection of DNA-drug interaction based on emission quenching of ZnS quantum dots via photoinduced electron transfer. *J. Lumin.* **2018**, 197, 112–118.
2. Bogdan, N.; Vetrone, F.; Ozin, G.A.; Capobianco, J.A. Synthesis of Ligand-Free Colloidally Stable Water Dispersible Brightly Luminescent Lanthanide-Doped Upconverting Nanoparticles. *Nano Lett.* **2011**, 11, 835–840.
3. Sapsford, K.E.; Berti, L.; Medintz, I.L. Materials for Fluorescence Resonance Energy Transfer Analysis: Beyond Traditional Donor-Acceptor Combinations. *Angew. Chem. Int. Ed.* **2006**, 45, 4562–4589.

4. Yordanov, G.; Skrobanska, R.; Evangelatov, A. Entrapment of epirubicin in poly(butyl cyanoacrylate) colloidal nanospheres by nanoprecipitation: Formulation development and in vitro studies on cancer cell lines. *Colloids Surf. B: Biointerfaces* **2012**, 92, 98–105.
5. Kaushik, D.; Saini, B.; Bansal, G. Identification of Four New Degradation Products of Epirubicin Through Forced Degradation, LC–UV, MSn and LC–MS–TOF Studies. *J. Chromatogr. Sci.* **2015**, 53, 1737–1748.

© 2019 by the authors. Submitted for possible open access publication under the terms and conditions of the Creative Commons Attribution (CC BY) license (http://creativecommons.org/licenses/by/4.0/).
